# Stability of dental implants with sandblasted and acid-etched (SLA) and modified (SLActive) surfaces during the osseointegration period

**DOI:** 10.34172/joddd.2021.037

**Published:** 2021-12-05

**Authors:** Gulsum Sayin Ozel, Ozgur Inan, Asli Secilmis Acar, Gamze Alniacik Iyidogan, Dogan Dolanmaz, Gulsun Yildirim

**Affiliations:** ^1^Department of Prosthodontics, Istanbul Medipol University, Istanbul, Turkey; ^2^Department of Prosthodontics, Selcuk University, Konya, Turkey; ^3^Department of Prosthodontics, Gaziantep University, Gaziantep, Turkey; ^4^Department of Prosthodontics, Mersin Dental Hospital, Mersin, Turkey; ^5^Department of Oral Maxillofacial Surgery, Bezmialem University, Istanbul, Turkey; ^6^Department of Oral Maxillofacial Surgery, Alaaddin Keykubat University, Antalya, Turkey

**Keywords:** Bone-implant interface, Dental implants, Osseointegration, Resonance frequency analysis, SLA, SLActive

## Abstract

**Background.** The surface properties of implants are effective factors for increasing the osseointegration and activity of osteoprogenitor cells. This study compared the stability of dental implants with sandblasted and acid-etched (SLA) and modified surfaces (SLActive) using the resonance frequency analysis (RFA).

**Methods.** In a split-mouth design, 50 dental implants with either SLA surface properties (n=25) or modified (SLActive) surface properties (n=25) were placed in the mandibles of 12 patients with a bilateral posterior edentulous area. Implant stability was measured using RFA (Osstell) at implant placement time and every week for 1, 2, and 3 months before the conventional loading time.

**Results.** One week following the implantation, implant stability increased from 70 to 77.67 for SLA and from 71.67 to 79 for SLActive (*P* < 0.05). Stability improved each week except in the 4th week in SLActive surface measurements. No significant differences were observed between the groups at 2 and 3 months (*P* > 0.05).

**Conclusions.** For both implant surfaces, increased stability was observed over time, with no significant differences between the groups.

## Introduction

Stability is a measure of the difficulty of disturbing an object or system’s equilibrium.^1^ Implant stability could be defined clinically as the capacity to resist rotation and axial-lateral loading without mobility. In implant dentistry, stability is an essential condition for treatment success.^2^ Long-term follow-up of dental implants has shown that maintaining implant stability improves the treatment success rate. Differences in implant designs and bone quality can affect implant stability.^3^ Clinical prognosis is determined by the relationship between bone quality, implant properties (i.e., length, surface roughness, and surface characteristics), and implant failure. Surgical trauma and anatomical conditions are the foremost factors in early implant failure, and factors for late implant failure include bone quality, the volume of bone, and overload.^4^

Consequently, the evaluation of implant stability is critical to ensure successful bone-implant contact. To date, many methods have been used to assess implant stability, such as insertion torque, sound upon percussion, anti-rotational torque, response to percussion (Perio-Test), and resonance frequency analysis (RFA). ^5^ However, RFA is most effective in evaluating implant stability during implant treatment and loading. Using RFA, implant stability can be gauged by quantifying the frequency of implant oscillation inside the bone. Changes in implant stability can be monitored during the implant healing process, and possible failure risks can be identified. RFA measurements are displayed as the implant stability quotient (ISQ), recorded as a number between 1 to 100; a higher ISQ value represents a higher degree of implant stability. ISQ values change from 40 to 80 for implants clinically described as stable.^6^

The implant surface is also a foremost factor in the osseointegration process.^7^ The prominent surface properties are topography, chemical properties, roughening, water contact angle, wettability, and hydrophilicity.^8^ Rough surfaces that provide a mechanical connection have been shown to augment the bone-implant contact surface quantity during the osseointegration process more than smooth surfaces. Implant surface properties also improve the osteogenic differentiation potential of osteoprogenitor cells. ^9^

Several techniques have been reported to modify surface properties and generate microroughness on titanium surfaces, including microindentation,^10^ acid-etching,^11^ a combination of sandblasting and acid etching,^12^ ion implantation,^13^ lasers,^14^ and surface oxidation.^15^ Among these techniques, sandblasted and acid-etched surfaces (SLA, Institute Straumann AG, Basel, CH) exhibited superior implant-bone contact than titanium plasma-sprayed, Al_2_O_3_-blasted, and polished implant surfaces in histomorphometric studies.^16^ SLA surfaces also displayed increased removal torque values in biomechanical testing. The SLA surface demonstrated improved osteoblast diﬀerentiation; production of osteogenic factors, cytokines, and growth factors; and increased implant-bone contact relative to smooth-machined surface implants.^17^ Previous studies reported the loading procedure of SLA surface implants after an early healing period of 6–8 weeks.^18^ Three-year clinical trials demonstrated success rates around 99­–100% and survival rates around 97.5%.^18-20^

Surface chemistry is another key variable for the predictability of the implant-bone response that influences implant-bone apposition.^21^ SLActive (SLActive, Institute Straumann AG, Basel, CH) implant surfaces (sandblasted, etched, and chemically modified), launched in 2005, have been enhanced to obtain early osseointegration and decrease the risks of early loading treatments, with properties such as enhanced precursor cell effectiveness and bone apposition. These properties are purported to enhanced stability and improve subepithelial connective tissue attachment during the preliminary osseointegration and early recovery period compared to SLA. SLA and SLActive surfaces are made of the same Grade 2 titanium and treated with the same sandblasting and acid-etching technique (250–500-μm corundum sandblasting + H_2_SO_4_/HCl acid etching). However, they differ because the SLActive surfaces have an additional procedure-laving under nitrogen conservation to avoid air contact and are kept in a sealed glass tube with an isotonic NaCl solution to prevent drying and preserve the clean TiO_2_ passivation layer for a more hydrophilic surface.^22^ Previous studies that compared the SLA and SLActive surfaces reported that SLActive surfaces provided 60% more bone formation and SLActive surfaces exhibited significant stability improvements two weeks after implant placement.^23^

Considering all this information, determining the differences between the SLA and SLActive implant surfaces during the 12-week follow-up period will be useful for implant selection. In contrast to earlier studies, in this study, SLA and SLActive surface implants were evaluated and compared with a split-mouth design over a 3-month period, which is the conventional healing period. Therefore, the present study aimed to compare the stability of implants with SLA and SLActive surface properties at the time of implant placement, and 1-, 2-, and 3-week, and 1-, 2-, and 3-month intervals following placement, using RFA.

## Methods

Twelve patients visiting Selcuk University Faculty of Dentistry, Oral and Maxillofacial Surgery Department, and Department of Prosthodontics, were recruited for the study. The patients were 20–50 years of age, had bilateral tooth loss in the mandible, and required rehabilitation with implant-supported fixed partial dentures. The study protocol was approved by the Ethics Committee of Selcuk University, Faculty of Medicine for human subjects, and written informed consent forms were obtained from all the participants. This clinical research complied with the Declaration of Helsinki on ethical principles. Patients with factors possibly affecting the results, such as systemic problems, pregnancy, heavy smoking, alcohol consumption, drug use, and anti-inflammatory agent or bisphosphonate use, were excluded. Dental implants were placed in the affected regions in the mandible with D2 or D3 bone quality. Tissue-level implants were used for implants with SLA and SLActive surfaces in this study. Eleven patients received four implants, and one patient received six implants, adding up to 50 implants. The implants were divided equally between SLA and SLActive surface implants, with one on each side. Clinical and radiographic examinations were completed in patients with the bilateral partially edentulous mandible. SLA-surface implants (Institute Straumann AG, Basel, Switzerland) were placed on one side, with SLActive-surface implants (Institute Straumann AG, Basel, Switzerland) on the other side.

Dental implants were positioned with a one-stage protocol. First, anesthesia (Ultracain DS; Aventis Pharmaceuticals, Istanbul, Turkey) was applied locally, and then mucoperiosteal flaps were removed. A total of fifty implants were placed in the SLA (n = 25) and SLActive (n = 25) groups in the mandible. Favorable implant-bone contact was achieved for every application. The mucoperiosteal ﬂaps were sutured with silk sutures (Sterisilk; SSM Sterile Health Products Inc, Istanbul, Turkey) with adaptation around the tissue-level dental implants’ cervical region, and healing caps (gingiva formers) were fitted.

Amoxicillin (500 mg, three times a day for 10 days), paracetamol (500 mg, twice a day for 5 days), and 0.2% chlorhexidine mouthwash (twice a day for 10 days) were prescribed after surgery. Ten-day follow-up appointments were scheduled to remove the sutures.

The implant stability (ISQ) of all the implants was measured using RFA (Osstell ISQ; Integration Diagnostics AB, Sävedalen, Sweden) throughout the healing period. First measurements were made before the healing cap was placed, followed by measurements 1 and 3 months after the surgical procedure. An Osstell probe was placed in both buccolingual and mesiodistal directions, and three measurements were obtained for all directions for both the SLA and SLActive groups. Average ISQ values were calculated, and all the values were ≥80 at 1- and 3-month intervals after surgery for all the implants.

RFA was used to compare the primary stability of the implants. For this purpose, the Osstell (Sävedalen Integration Diagnostics, Switzerland) equipment was used. A Smartpeg was screwed with 4–5-Ncm torque on the implants. When the probe was kept almost touching the Smartpeg, the instrument displayed the value of an audio transmitter, and the ISQ value was recorded. Measurements made immediately after implant placement (0) were repeated at 1-, 2, and 3-week and 1-, 2-, and 3-month intervals. Data were analyzed with repeated-measures ANOVA using SPSS 21.0 (SPSS Inc., Chicago, IL, USA) at a significance level of *P* = 0.05.

## Results

**Figure 1:**
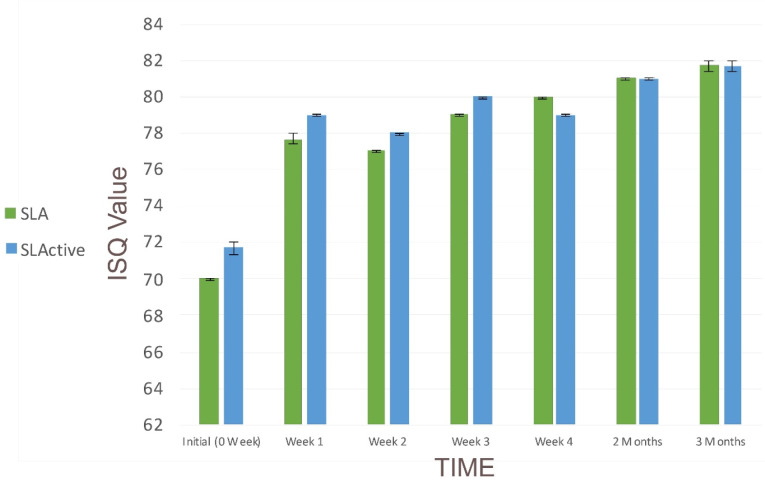
ISQ values of implants with SLA and SLActive surfaces.

Implant mobility and peri-implant infection were not observed during the healing period. The changes in the mean ISQ values of SLA and SLActive surfaces over time are presented in Table 1 and Figure 1. After implantation, the ISQ values increased from 70 to 77.67 for SLA and from 71.67 to 79 for SLActive after one week (*P* < 0.05). SLActive surface measurements increased each week except the 4th week, in which ISQ decreased (*P* < 0.05). No significant differences were observed between the groups at 2- and 3-month intervals (*P* > 0.05). For both implant surfaces, an increase was observed over time, with no significant differences between the groups.

**Table 1:** RFA measurements from implant placement time (initial time) during the osseointegration period

**Time (wk)**	**ISQ Values**	
	**SLA**	**SLActive**
0	70.00 ± 0.00^g^	71.67 ± 0.33^f^
1	77.67 ± 0.33^de^	79.00 ± 0.00^c^
2	77.00 ± 0.00^e^	78.00 ± 0.00^d^
3	79.00 ± 0.00^c^	80.00 ± 0.00^b^
4	80.00 ± 0.00^b^	79.00 ± 0.00^c^
8	81.00 ± 0.00^a^	81.00 ± 0.00^a^
12	81.67 ± 0.33^a^	81.67 ± 0.33^a^

Note: The same superscript indicates a statistically insignificant difference. repeated measurements ANOVA (*P* < 0.05).

Table 2 presents the mean stability values of implants with SLA and SLActive surfaces, statistical analysis results, and the comparisons between the two groups. There were no significant differences between SLA and SLActive surfaces in terms of stability (*P* > 0.05).

**Table 2:** Mean ISQ values and comparison of SLA and SLActive implant surfaces

**Surface type**	**Mean**	**Std. error**	**95% Confidence interval**		***P*** ** value**
			**Lower bound**	**Upper bound**	
SLA	78.084	1.052	75.954	80.213	0.763
SLActive	78.536	1.052	76.407	80.665	0.763

## Discussion

In the present study, the stability of two different implant surfaces was evaluated and compared at baseline, 1-week, 2-week, 3-week, 1-month, 2-month, and 3-month postoperative intervals, using RFA with a randomized controlled split-mouth design. The split-mouth design provided the standardization of the bone quality and systemic effect and eliminated the probable impact of patient biotype and/or lifestyle on the outcomes. This design also allows us to control the patient’s bone healing capacity, general health, and hygiene habits.^24^ Implant primary stability is significant for the evaluation of implant survival and success rate. Gupta and Padmanabhan argued that primer stability is the most important mechanical factor for implant osseointegration success. In addition, primer stability is associated with bone density, bone type, implant morphology, and surgical and loading procedures.^25^ For this reason, patients with type II bone density, no systemic problems, and no necessity for grafting were included in the present study. In addition, the mandible was preferred for this study as primary stability in the mandible is more effective than that in the maxilla.^26^ The main limitation of the current study was the recruitment of Kennedy Class I patients. Thus, the main results might be generalized only to the posterior mandible. The reason for our participant selection scheme was to standardize bone regions to ensure bone homogeneity and to eliminate confusing factors such as grafting or non-standardized bone quality.

RFA is currently the most favored non-invasive method for implant stability. Non-invasiveness, ease of implementation, repeatability of measurements at every stage, and clinically similar stability values are some of the advantages of the RFA method compared with other methods of implant stability measurement.^27,28^ Many studies have reported using the RFA method, similar to our study.^29,30^ In this study, the Osstell Mentor was used to evaluate stability. The advantages of the Osstell Mentor include its easy use, clinical repeatability of measurements, and its non-invasive application. Thus, the Osstell Mentor can be used to monitor the prognosis of implants and evaluate the conformity of the implant for loading protocols.^31^ Similarly, we also used the RFA to measure implant stability during the osseointegration period.

Rough titanium surfaces clinically induce rapid osseointegration, compared with smooth surfaces, while ensuring the maximum bone-implant contact and blood cell migration and differentiation to the precursor osteogenic cells. The roughness value of the SLA surface is nearly 2.93 mm, with a range of 1.2–3.9 mm for the SLActive surface, which undergoes the same roughening procedure. Accordingly, SLA and SLActive implants have a similar surface topography. One study concluded that rougher surfaces represent more bone-implant contact and that the chemical properties of surfaces that affect wettability might also affect bone migration to the implant surface.^32^ Chemically modified, sandblasted, large-grit, and acid-etched (SLActive) titanium surfaces have been developed to improve surface wettability. Hydroxylated/hydrated SLActive implants have an initial advancing water contact angle of 0°, and SLA implants have a 138–140° advancing water contact angle,^33^ which provides the SLActive implants with a more hydrophilic surface than SLA implants. More studies reported that SLActive surfaces exhibited optimal osseointegration by transforming their hydrophobic surface to a hydrophilic surface, producing a nano rough surface. When the implants contact blood, the ultrahydrophilic surface reactivates, and an indiscrete conditioning stratum occurs. Some studies demonstrated that the SLActive surface has a greater bone-implant contact at 2 and 4 weeks compared with the SLA surface. ^34,35^

Han et al^36^ evaluated the effect of SLA and SLActive surface type and diameter on implant stability according to ISQ values, concluding that there were no significant differences in ISQ values after three months. These results consisted of bone shape and remodeling at the initial stage.^37^ Similarly, during the three-month follow-up in the present study, favorable primary and secondary stability was observed for both surface types with ISQ values >70. Similar to the present study’s results, Oates et al^38^ found that implant stability increased after two weeks for the SLActive implant and four weeks for the SLA implant. Another clinical comparison of SLA and SLActive implants reported a higher survival rate for the SLA implants.^39^ In contrast, a three-year follow-up study showed a lower success rate for the SLA implants than SLActive implants.^18^ A recent comparative histological study with SLA and SLActive implants reported that the use of an activated implant surface did not increase bone-implant contact compared to conventional implants.^40^ Additionally, a recent review that included 1394 SLA and 145 SLActive implants demonstrated no significant differences concerning implant loss or clinical parameters between the immediate/early loading and late loading protocols.^41^ This finding is similar to those of the current study, which compared and evaluated the stability of implants with SLA and SLActive surfaces clinically at 0, 1, 2, 3, 4, 8, and 12 weeks with the RFA method. There was little difference at 0, 1, 2, and 3 weeks for SLActive implants and a slight decrease at four weeks, but these differences were not statistically significant. At 8–12 weeks, SLA and SLActive values were identical to each other. These similar values might be explained by perfect primary stability. In addition, according to the weekly comparison, at week 0, the values were significantly lower than at other intervals, and the stability values increased each week for both SLA and SLActive implants.

## Conclusion

Within the limitations of this study, it can be concluded that if the bone quality is good, like in the mandible, the SLActive surface does not have any advantages over the SLA surface, and we suggest more split-mouth studies with exact site-specific matching.

## Limitations

This study had certain limitations. First, three months is a relatively short time for evaluating primary efficacy. However, in this study, the main target was to evaluate the osseointegration phase each week for both implant surfaces. In addition, different surface types, both the mandible and maxilla osseointegration period, and different conditions that might affect the osseointegration could be investigated in future studies.

## Authors’ Contributions

GSO, OI, and ASA contributed to the study concept and design. GAI, DD,and GY contributed to material preparation and data collection and analysis. All the authors commented on previous versions of the manuscript. All the authors read and approved the final manuscript.

## Acknowledgments

None.

## Funding

Selcuk University Scientific Research Projects Committee funded this study.

## Competing Interests

The authors declare no conflict(s) of interest related to the publication of this work.

## Ethics Approval

The Ethics Committee of Selcuk University approved the protocol of the study under the code 2006/06.
